# Associations between Dietary Intake and Urinary Bisphenol A and Phthalates Levels in Korean Women of Reproductive Age

**DOI:** 10.3390/ijerph13070680

**Published:** 2016-07-05

**Authors:** Ara Jo, Hyesook Kim, Hyewon Chung, Namsoo Chang

**Affiliations:** 1 Department of Nutritional Science and Food Management, Ewha Womans University, 52, Ewhayeodae-gil, Seodaemun-gu, Seoul 03760, Korea; noelle5@naver.com (A.J.); khs7882@hanmail.net (H.K.); 2 Department of Obstetrics and Gynecology, Ewha Womans University School of Medicine, 1071, Anyangcheon-ro, Yangcheon-gu, Seoul 07985, Korea; hyewon@ewha.ac.kr

**Keywords:** beverage, Bisphenol A, dietary intake, egg, phthalates, reproductive aged women

## Abstract

Human exposure to Bisphenol A (BPA) and phthalates is a growing concern due to their association with harmful effects on human health, including a variety of disorders of the female reproductive system. The objective of this study was to investigate the association between food intake and urinary BPA and phthalates in Korean women of reproductive age. A cross-sectional study was conducted with 305 reproductive aged (30–49 years) females in Korea. Dietary intake was assessed using 24 h dietary recall, and urinary BPA and particular phthalates were measured using high performance liquid chromatography tandem mass spectrometry. After adjusting for covariates, beverage intake was positively associated with urinary BPA, and egg and egg product intake was negatively associated with urinary mono-*n*-butyl phthalate (MnBP) as well as mono (2-ethyl-5-oxohexyl) phthalate (MEOHP). Odds ratio for high BPA level (≥90th percentile) in women with >100 g of beverage consumption was significantly higher than for those who consumed ≤100 g. These results suggest that, in Korean women of reproductive age, some foods such as beverages and egg may be associated with body burdens of BPA, MnBP, MEHHP and MEOHP.

## 1. Introduction

Human exposure to Bisphenol A (BPA) and phthalates is a growing concern due to their harmful effects on human health including a variety of disorders of the female reproductive system [1,2,3,4,5,6,7,8,9]. Some studies have reported that dietary intake is associated with urinary BPA and phthalates [10,11,12]. BPA is widely used in the manufacture of polycarbonate (PC) plastics, epoxy resins, and polyvinyl chloride (PVC), which are commonly used as plastic and metal materials used for food and drink packaging [13,14,15,16]. Phthalates are also added to PVC food containers to increase their flexibility and durability [16]. Previous studies have found that increased intake of foods packaged in materials containing BPA and phthalates led to increased concentrations of these compounds in urine [10,17].

Human urinary BPA and three phthalate metabolites—mono-*n*-butyl phthalate (MnBP), mono (2-ethyl-5-hydroxyhexyl) phthalate (MEHHP), and mono (2-ethyl-5-oxohexyl) phthalate (MEOHP)—have been detected in >90% of individuals from representative Korean populations [18,19]. Similar results were shown by the US National Health and Nutrition Examination Survey (NHANES) 2003–2004 [20,21], and in studies on some Asian populations, including China, India, Japan, Korea, Kuwait, Malaysia, and Vietnam [22].

Recently, the use of BPA and some phthalates in food containers has been restricted in the European Union [23], however, some studies reported the migration of BPA and phthalates from food containers into the foods and beverages packaged therein [24,25,26,27]. Laboratory studies have determined that the concentration of BPA was significantly higher in beverages from canned or polycarbonate bottles than for similar beverages in other containers [25,27]. Intervention studies found that urinary BPA concentration was significantly higher after consuming beverages from cans or polycarbonate drinking bottles compared to the concentrations before consumption [28] or after consuming beverages from glass containers [29]. Dietary intake is a significant route for phthalate exposure, and some foods, such as poultry, dairy products, meat, and seafood, have also been associated with increased phthalate body burden [12,17].

BPA and phthalates are endocrine disruptor chemicals that are commonly referred to as environmental estrogens, neuroendocrine disruptors, or biological disruptors, and may alter the estrogen receptor function by blocking or mimicking the action of estrogen [9,30]. An increasing number of studies indicate that human exposure to BPA and phthalates can lead to impairment of the female reproductive system [1,2,3,4,5,6,7,8,9] and have health effects on the second generation [31,32,33].

Although many studies have demonstrated an association between the some food intake and body burden of BPA and some phthalates, no studies have investigated such a relationship in reproductive aged women. Given that BPA and some phthalates are risk factors for disorders in the female reproductive system, and that its exposure level can be varied by diet, studies on the relationship between BPA and some phthalate metabolites and diet may help develop core strategies for dietary intervention strategies. Therefore, in this study the association between dietary food intake and the urinary concentrations of BPA and metabolites of di(2-ethylhexyl)phthalate (DEHP) and di-*n*-butyl phthalate (DBP) was investigated in Korean women of reproductive age.

## 2. Methods

### 2.1. Study Population

The study population comprised 307 married childbearing women between the ages of 30 and 49 years, recruited from Seoul, Republic of Korea in May 2014. Each participant provided written consent. Subjects who had food energy consumption less than 500 kcal/day (*n* = 2) were excluded from the study, leaving 305 eligible subjects. The Institutional Review Board (IRB) at Ewha Womans University Hospital in Seoul, Republic of Korea, reviewed and approved the study protocol (IRB number 2014-06-014-003).

### 2.2. General Characteristics and Anthropometric Parameters

Participants were personally interviewed, including questions on age; education; occupation; and health habits such as cigarette smoking, passive smoking and alcohol consumption status. Standing height was measured using automatic instrument (DS-102; Dong Sahn Jenix, Seoul, Korea), body weight (kg) and body fat (kg) were measured using INBODY 230 (Biospace Co., Seoul, Korea), and body mass index (BMI = weight (kg)/height squared (m^2^)) was calculated. Waist circumference was measured midway between the lowest rib and the iliac crest with a tapeline (Anthropometric tape, Preston 5193, Seoul, Korea).

### 2.3. Dietary Assessment

Dietary intake was assessed on the day before urine sampling using single 24 h dietary recall by trained dietary interviewers. Participants were asked to complete a dietary survey on all the foods and beverages they had consumed over the last 24 h before the interview. Three days after the interview, follow-up was made by phone to ascertain detailed information on the types of food and drinks consumed, portion size and extra condiments. Canned and sachet coffee, plastic and can packed tea, soda and alcohol were included as beverages. Food intake data were analyzed using the computer aided nutritional analysis program Can-Pro (Can-Pro 4.0, Nutritional Assessment Program, 2011, The Korean Nutrition Society, Seoul, Korea).

### 2.4. BPA and Phthalate Metabolite Measurements

We measured BPA and the metabolites of DEHP and DBP among several phthalate compounds using a method combining liquid-liquid extraction and liquid chromatography tandem mass spectrometry (HPLC-MS/MS) (Model 6410b, Agilent Technologies, Santa Clara, CA, USA). DEHP is metabolized to MEHHP and MEOHP, and DBP is metabolized to MnBP. Approximately 5–10 mL of morning spot urine were collected from each participant at 8:30 A.M., then frozen and stored at −20 °C before analysis.

#### 2.4.1. Determination of BPA in Urine

The determination of urinary BPA was performed as descried by Matsumoto et al. [11], with minor modifications. In brief, for the analysis of total urinary BPA (conjugated and free form), 30 µL of 2.0 M sodium acetate (pH 5.0), and 20 μL β-glucuronidase (Roche, Indianapolis, IN, USA) were added to 1 mL aliquots of each urine sample in a 15 mL glass tube. The reaction mixture was incubated at 37 °C for 16 h. After the incubation, we added 100 μL of 2 N HCl and extracted the mixture with 3 mL of ethyl acetate that included 100 ng/mL BPA in acetonitrile as an internal standard. After the extraction, we transferred 2 mL of supernatant to a new glass tube and evaporated the solution. The residue was dissolved in 300 μL of 60% acetonitrile and 5 μL of this resultant was injected into our HPLC-ESI-MS/MS apparatus. The HPLC column used was Agilent Eclipse plus C18 3.5 μm (2.1 mm × 100 mm), which was maintained at 35 °C.

#### 2.4.2. Determination of MnBP, MEHHP and MEOHP in Urine

The determination of urinary MnBP, MEHHP and MEOHP were performed as descried by the CDC Phthalate Metabolite Laboratory Procedure Manual [34], with minor modifications. In brief, for the analysis of urinary phthalate metabolite, 30 μL of 2.0 M sodium acetate (pH 5.0), and 20 μL β-glucuronidase (Roche) were added to 1 mL aliquots of each urine sample present in a 15 mL glass tube. The reaction mixture was incubated at 37 °C for 16 h. After the incubation, we added 100 μL of 2 N HCl and extracted the mixture with 3mL of ethyl acetate that included 100 ng/mL MnBP, MEHHP and MEOHP in acetonitrile as an internal standard. After the extraction, we transferred 2 mL of supernatant to a new glass tube and evaporated the solution. The residue was dissolved in 300 μL of 60% acetonitrile and 5 μL of this resultant was injected into our HPLC-ESI-MS/MS apparatus. The HPLC column used was a Chromolith Performance RP-18e 3.5 μm (3 mm × 100 mm), which was maintained at 35 °C.

#### 2.4.3. Validation Procedure

Linearity in these analytes was checked with correlation coefficients of 0.997 and 0.999 for BPA and phthalates, respectively. Recovery tests were performed by adding known amounts of the standards and gave values of 87.4%–109.0% for BPA and 85.9%–108.8% for phthalates. The intra- and inter-day accuracy and precision were investigated by determining the four analytes in the seven replicates during a single day and by duplicating the experiments during five consecutive days. The intra-day accuracy for BPA was 91.2%–114.5% with a precision of 5.2%–11.2%, whereas its inter-day accuracy was 96.4%–108.4% with a precision of 3.8%–6.7%. The intra-day accuracy of phthalates was 96.4%–110.5% with a precision of 5.6%–10.2%, whereas its inter-day accuracy was 97.8%–106.4% with a precision of 3.2%–8.1%. Variations of the measured concentrations of these standards were between 10% and 15% depending on the metabolite. External quality assurance was provided by the German External Quality Assessment Scheme for Biological Monitoring (G-EQUAS). The HPLC MS/MS was operated in the negative ESI multiple reaction monitoring mode (MRM) with the following source parameters: nitrogen gas temperature 340 °C, gas flow 10 L/min, nebulizer 40 psi, capillary 2500 volts. The MRM transitions monitored were: *m*/*z* 227→133 for BPA, *m*/*z* 221.2 → 77.1 for MnBP, *m*/*z* 293.2 → 121.0 for MEHHP, and *m*/*z* 291.2 → 120.8 for MEOHP. The ranges of the calibration curve were 2–200 μg/L for BPA and MnBP, and 2–100 μg/L for MEHHP and MEOHP). The limit of quantification (LOQ) and the limit of detection (LOD) for each analyte under the chromatographic conditions were determined at the signal-to-noise ratio (S/N) of 3 and 10, respectively. The LOQ for urinary BPA, MnBP, MEHHP and MEOHP were 0.4, 1.0, 0.8, and 0.7 μg/L, respectively. The LOD for urinary BPA, MnBP, MEHHP, and MEOHP was 0.1, 0.3, 0.2, and 0.2 μg/L, respectively. Individuals whose urinary concentration fell below the LOD were assigned a value of LOD/2 [35]. Urinary creatinine concentration was assessed using the Jaffe reaction [36] on a Siemens Advia 1800 analyzer (Siemens, Healthcare Diagnostics, Deerfield, IL, USA). BPA, MnBP, MEHHP and MEOHP concentrations were adjusted for urinary creatinine concentration to correct for the urine volume.

### 2.5. Statistical Analysis

Continuous values were described as means with standard deviations (SD) and categorical values were represented as frequency and percentage of subjects. Urine profiles were log transformed to normalize their distributions. A partial correlation coefficient was used to assess the correlation of urinary BPA, MnBP, MEHHP and MEOHP with food intake after adjustments for age, BMI, total energy intake, education, passive smoking, and alcohol consumption. Multiple regression analysis was used to examine the relationship between urinary levels of BPA, MnBP, MEHHP and MEOHP and dietary food intake. Binary logistic regression analysis with adjusted models controlling for confounders was also used to identify the odds ratios (ORs) and 95% confidence intervals (CIs) for high BPA levels (≥90th percentile) depending on the beverage intake (≤100 g/day or >100 g/day). All statistical analyses were performed using SAS 9.4 (SAS Inc., Cary, NC, USA). The significance level was defined as *p* < 0.05.

## 3. Results

Table 1 shows summary statistics for the general characteristics. The mean age of the study population was 36.8 ± 4.4 years, BMI was 22.3 ± 3.1 kg/m^2^, waist circumference was 76.5 ± 8.1 cm and body fat was 18.0 ± 5.8 kg.

Table 2 shows population mean urine concentrations for the target metabolites. The mean values for urinary BPA was 1.7 ± 1.5 μg/g creatinine, and the means of urine MnBP, MEHHP and MEOHP were 41.0 ± 48.1 μg/g creatinine, 13.9 ± 19.2 μg/g creatinine and 9.8 ± 13.3 μg/g creatinine, respectively.

Table 3 shows the mean dietary statistics for the study population. The average total food intake was 966.4 ± 351.8 g/day. The means for fruits and vegetables, beverages, and eggs and egg products consumption was 308.7 ± 201.8 g/day, 115.5 ± 159.9 g/day and 22.8 ± 30.1 g/day, respectively.

Table 4 shows the correlations between urinary BPA and phthalate metabolites. Urinary BPA is positively correlated with BMI (*r* = 0.1744, *p* = 0.0022), waist circumference (*r* = 0.1890, *p* ≤ 0.001), body fat (*r* = 0.1884, *p* ≤ 0.001) (data not shown), and beverage consumption (*r* = 0.1616, *p* = 0.0051); and negatively correlated with fruits and vegetables intake (*r* = −0.1257, *p* = 0.0298), after adjustment for age, BMI, total energy intake, education, passive smoking, and alcohol consumption. Egg and egg product intake is inversely correlated with urinary MnBP (*r* = −0.1231, *p* = 0.0333) and MEOHP (*r* = −0.1157, *p* = 0.0456) after adjustment for covariates.

Table 5 shows the coefficients from multiple regression analysis of the trial data. Beverage intake is positively associated with urinary BPA after adjustment for covariates (*β* = 0.0007, *p* = 0.0118), whereas Egg and egg product intake is negatively associated with urinary MnBP (*β* = −0.0029, *p* = 0.0342) and MEOHP (*β* = −0.0034, *p* = 0.0459) after adjustment for covariates.

Table 6 shows multiple logistic regression analysis of the trial data. The OR of high BPA level (≥ 90th percentile) in women with >100 g beverage consumption [OR = 2.374; 95% CI = 1.081–5.215] was significantly higher than those with ≤100 g consumption.

## 4. Discussion

Human exposure to BPA and phthalates in relation to food consumption has been investigated in other many studies, but to our knowledge, this is the first such study on Korean reproductive aged women.

The major finding from the present study was a positive association between dietary beverage consumption and urinary BPA. Similar results have been reported from cross-sectional studies in other countries. NHANES 2005–2006 demonstrated soda intake had a positive association with urinary BPA in a representative US population [10], and urinary BPA concentration was positively correlated with beverage intake, such as coffee and tea, for 50 university students in a Japanese study [11]. Some intervention studies also indicated migration of BPA to the contents from the beverage containers. Bae et al. [29] reported increased urinary BPA concentrations in Koreans aged ≥ 60 years who consumed canned beverages compared with consumption of the same beverages from a glass bottle in a randomized crossover trial. A previous non-randomized intervention study found higher urinary BPA levels after participants consumed cold beverages from polycarbonate drinking bottles when compared with levels before consumption [28]. The particular beverages investigated here were canned and sachet coffee, plastic and can packed tea, soda, and alcohol. BPA is primarily used in the manufacture of polycarbonate plastics and epoxy resins commonly used in plastics and canned food containers [37,38,39,40,41,42], which come into direct contact with the foods and beverages and transfer BPA to the contents [25,26,43,44]. Although, the packaging materials used for the beverages were not investigated here, many studies have revealed that the concentrations of BPA were higher in canned and polycarbonate plastic container beverages than in similar beverages in other containers [25,26,27].

The present study found a weak negative association between egg and egg product intake and urinary MnBP and MEOHP. Colacino et al. [45] found that consumption of poultry as well as eggs or egg products was positively associated with urine mono-2-ethylhexyl phthalate (MEHP) levels, which is one of the phthalate metabolites, in representative US participants. This suggests that chickens themselves may be contaminated with phthalates and food is being contaminated not only through packaging and processing. A similar result to the current trial finding was recently reported from an investigation of Chinese children (8–16 years), where egg consumption was negatively correlated with four phthalate metabolites, including MEHHP and MEOHP [12]. Dietary egg or egg product consumption was not associated with beverage consumption in the current trial (data not shown). However, lower egg intake was associated with greater urinary levels of MnBP and MEOHP. Thus, the association was not that egg consumption lead to less other food consumption, such as beverages, which were packed in plastic containers. There is no apparent literature providing a scientific explanation for this finding. Therefore, future studies are warranted to identify the underlying mechanisms.

The present study found a weak negative correlation between fruit and vegetable intake and urinary BPA concentrations in partial correlation analysis but not in multiple regression analysis. In a previous intervention study, a group of 20 participants in the US had 66 percent less urinary BPA concentrations after 3 days on a diet of fresh and organic vegetables, fruits, grains, and meats that were not packaged in cans or plastic [46]. Other cross-sectional studies have reported that fruit and vegetable intake was negatively associated with urinary BPA concentrations, but those results were not significant [47,48]. Fruit and vegetable intake was negatively associated with beverage intake in the current trial (data not shown), which indicates that the weak negative correlation may be related to that fruit and vegetable consumption lessening the consumption of food, e.g., beverages, packed in cans or plastic containers.

The mean level of urinary BPA (1.7 ± 1.5 μg/g creatinine) was comparable to that found in pregnant Korean women [32], a Korean female aged 73.1 years [29], and Asian populations (mean age 32 years) including Korea, China, Vietnam, Malaysia, India, Kuwait and Japan [22], as well as the general US population [49]; but lower than that reported in European countries [50]. The mean concentrations of urinary MnBP (41.0 ± 48.1 μg/g creatinine), MEHHP (13.9 ± 19.2 μg/g creatinine), and MEOHP (9.8 ± 13.3 μg/g creatinine) were similar or slightly lower than those found in female populations from Korea [19], China [51], US [49], and The Netherlands [52]. The reason for the relatively minor discrepancies among these data is unclear, but may be related to significant demographic differences between the subjects, including their ethnicity, age and pregnancy state.

Exposure to BPA and phthalates can cause impairment of female reproductive system. An increasing number of animal and human studies have indicated that BPA and phthalates exposure is associated with polycystic ovary syndrome [1,2], recurrent miscarriages [3], many reproductive indices [4,5,6,8] and health effects in the second generation [31,32,33]. In Korean women of reproductive age, beverage consumption has increased greatly in recent times, with alcohol consumption increasing approximately twofold from 118.0 g/day in 2008 to 273.6 g/ day in 2014 in females 30–49 [53,54]. Considering the harmful effects of exposure to BPA and phthalates on the female reproductive system, there have been heightened concerns about these increasing trends for reproductive aged Korean women in particular.

Several limitations of this study should be noted. The trial was cross-sectional, hence, the association between dietary intake and urinary BPA and phthalates metabolites levels is unclear whether dietary intake was a cause or consequence of increased urinary BPA and phthalates metabolites concentrations. The sample size (*n* = 305) may not be large enough to identify whether the beverage intake was related to urinary BPA concentration, and may not represent the general population. Although food containers are the main source of BPA and phthalates exposure, an examination of the packaging materials was not conducted, and the exact source of BPA and phthalates metabolites is not reflected in this study. In this regard, daily bottled water consumption, which can be a major source of BPA exposure, was not noted in the dietary recall interview. Spot urine samples were collected, and the exact time of the day was unavailable, making it difficult to assess the effect of time of urine collection on BPA and some phthalate metabolites concentration. Moreover, there is concern as to whether single spot urine tests accurately represent long term prenatal exposure to BPA and phthalate metabolites, because they have short half-life (4–24 h). Dietary intake assessed using a single 24 h recall may be insufficient to estimate the normal daily intake and represent a short term food intake. However, trained dietary nutritionists conducted direct face to face interviews and minimized potential errors when assessing dietary consumption. Other research has also shown that the values for total energy and other nutrients were not significantly different between subsequent 24 h dietary recalls (1.1% for energy) [55].

On the other hand, although it is well known that BPA and phthalates are risk factors for disorders in the female reproductive system, and that the major source of BPA and particular phthalates exposure is dietary intake, no prior study has been published on the association between dietary food intake and urinary concentrations of BPA and some phthalates metabolites in reproductive aged women.

## 5. Conclusions

A positive association was identified between beverage intake and urinary BPA, and a weaker negative association between egg intake and MnBP and MEOHP in Korean women of reproductive age. A larger scale follow-up study is required with improved methodology for urine sampling and food consumption questionnaire to provide data relevant for daily excretion and intake. Further studies are also warranted to explore the mechanisms underlying the association between these foods and urinary BPA, MnBP, MEHHP and MEOHP concentrations.

## Figures and Tables

**Table 1:** Study population general characteristics ^a^ (*n* = 305).

Characteristics	Values
Age (year)	36.8 ± 4.4
Height (cm)	160.4 ± 5.8
Weight (kg)	57.3 ± 7.9
Body mass index (kg/m^2^)	22.3 ± 3.1
Waist circumference (cm)	76.5 ± 8.1
Body fat (kg)	18.0 ± 5.8
Education	
<University	87 (28.5)
≥University	218 (71.5)
Occupation	
Housewives	204 (66.9)
Employed	101 (33.1)
Smoking status	
No	300 (98.4)
Yes	5 (1.6)
Passive smoking	
No	142 (46.6)
Yes	163 (53.4)
Alcohol use	
No	97 (31.8)
Yes	208 (68.2)

^a^ Values are mean ± SD or frequency(%).

**Table 2:** Study population urinary BPA and phthalates concentrations ^a^ (*n* = 305).

Chemical (μg/g Creatinine)	Values	Range
BPA	1.7 ± 1.5	0.1–18.3
MnBP	41.0 ± 48.1	1.8–527.9
MEHHP	13.9 ± 19.2	0.7–200.0
MEOHP	9.8 ± 13.3	0.5–137.9

^a^ Values are mean ± SD; Abbreviations: BPA = Bisphenol A, MnBP = mono-*n*-butyl phthalate, MEHHP = mono-(2-ethyl-5-hydroxyhexyl) phthalate, MEOHP = mono-(2-ethyl-5-oxohexyl) phthalate.

**Table 3:** Study population dietary intakes ^a^ (*n* = 305).

Food Group	Intake (g/day)
Total food intake	966.4 ± 351.8
Total plant food	756.4 ± 315.6
% Total plant food	78.0 ± 13.6
Cereal and cereal products	236.1 ± 109.5
Potatoes and starch products	40.2 ± 75.1
Fruits and vegetables	308.7 ± 201.8
Vegetables	195.4 ± 116.1
Fruits	113.3 ± 166.3
Seaweeds	2.7 ± 9.6
Mushrooms	5.4 ± 13.8
Soy beans and bean products	24.1 ± 44.0
Nut seeds and products	8.5 ± 29.6
Beverages	115.5 ± 159.9
Sugar and sugar products	7.1 ± 9.1
Fats and oils	8.1 ± 5.9
Total animal food	210.0 ± 145.9
% Total animal food	22.0 ± 13.6
Eggs and egg products	22.8 ± 30.1
Meats and meat products	66.0 ± 71.1
Fish and fish products	41.8 ± 47.0
Milk and milk products	79.4 ± 125.5

^a^ Values are mean ± SD.

**Table 4:** Pearson’s partial correlation coefficients between food intake and urinary BPA and phthalates concentration ^a^ (*n* = 305).

		BPA		MnBP		MEHHP		MEOHP	
		r	*p*	r	*p*	r	*p*	r	*p*
Fruits and vegetables	r1	−0.0928	0.1056	−0.0008	0.9887	0.0920	0.1088	0.0984	0.0862
	*r2*	−0.1164	**0.0432**	−0.0216	0.7089	0.0648	0.2619	0.0633	0.2726
	*r3*	−0.1257	**0.0298**	−0.0343	0.5542	0.0658	0.2565	0.0643	0.2674
Eggs and egg products	r1	−0.0872	0.1284	−0.1189	**0.0380**	−0.0946	0.0991	−0.1048	0.0677
	*r2*	−0.0966	0.0939	−0.1218	**0.0344**	−0.1047	0.0692	−0.1173	**0.0416**
	*r3*	−0.0933	0.1075	−0.1231	**0.0333**	−0.1023	0.0774	−0.1157	**0.0456**
Beverages	r1	0.1683	**0.0032**	0.1132	**0.0483**	0.0226	0.6945	0.0356	0.5362
	*r2*	0.1646	**0.0041**	0.1013	0.0790	−0.0031	0.9577	0.0055	0.9242
	*r3*	0.1616	**0.0051**	0.1071	0.0645	−0.0154	0.7910	−0.0060	0.9184

^a^ Urine profiles are log transformed; r1: Pearson’s correlation analysis; r2: Partial correlation analysis adjusted for age, BMI and total energy intake; r3: Partial correlation analysis adjusted for age, BMI, total energy intake, education, passive smoking and alcohol drink; Abbreviations: BPA = Bisphenol A, MnBP = mono-*n*-butyl phthalate, MEHHP = mono-(2-ethyl-5-hydroxyhexyl) phthalate, MEOHP = mono-(2-ethyl-5-oxohexyl) phthalate.

**Table 5:** Coefficients from multiple regression analysis between food intake and urinary BPA and phthalates concentration (*n* = 305).

	**BPA**		
	***β***	**(SE)**	***p***
Model 1			
Fruits and vegetables intake	−0.0003	0.0002	0.1282
Beverages intake	0.0007	0.0003	**0.0038**
Model 2			
Fruits and vegetables intake	−0.0004	0.0002	0.1029
Beverages intake	0.0007	0.0003	**0.0092**
Model 3			
Fruits and vegetables intake	−0.0004	0.0002	0.0727
Beverages intake	0.0007	0.0003	**0.0118**
	**MnBP**		
	***β***	**(SE)**	***p***
Model 1			
Eggs and egg products intake	−0.0028	0.0013	**0.0348**
Beverages intake	0.0005	0.0003	**0.0442**
Model 2			
Eggs and egg products intake	−0.0029	0.0014	**0.0369**
Beverages intake	0.0004	0.0003	0.0847
Model 3			
Eggs and egg products intake	−0.0029	0.0013	**0.0342**
Beverages intake	0.0005	0.0003	0.0661
	**MEOHP**		
	***β***	**(SE)**	***p***
Model 1			
Eggs and egg products intake	−0.0030	0.0017	0.0668
Beverages intake	0.0002	0.0003	0.5193
Model 2			
Eggs and egg products intake	−0.0034	0.0017	**0.0421**
Beverages intake	0.0000	0.0003	0.9569
Model 3			
Eggs and egg products intake	−0.0034	0.0017	**0.0459**
Beverages intake	0.0000	0.0003	0.9015

Model 1: Unadjusted; Model 2: Adjusted for age, BMI and total energy intake; Model 3: Adjusted for age, BMI, total energy intake, education, passive smoking and alcohol drink; Abbreviations: BPA = Bisphenol A, MnBP = mono-*n*-butyl phthalate, MEHHP = mono-(2-ethyl-5-hydroxyhexyl) phthalate, MEOHP = mono-(2-ethyl-5-oxohexyl) phthalate.

**Table 6:** Odds ratio (OR) and 95% confidence interval (CI) of urinary BPA levels ≥90th percentile (3.0 μg/g creatinine) according to beverage intake (*n* = 305).

	OR	95% CI
Model 1		
Beverage intake		
≤100 g (*n* = 204)	1 (ref.)	
>100 g (*n* = 101)	2.721	**1.278–5.791**
Model 2		
Beverage intake		
≤100 g (*n* = 204)	1 (ref.)	
>100 g (*n* = 101)	2.515	**1.159–5.458**
Model 3		
Beverage intake		
≤100 g (*n* = 204)	1 (ref.)	
>100 g (*n* = 101)	2.374	**1.081–5.215**

Model 1: Adjusted for fruits and vegetables intake; Model 2: Adjusted for fruits and vegetables intake, age, BMI and total energy intake; Model 3: Adjusted for fruits and vegetables intake, age, BMI and total energy intake education, passive smoking and alcohol drink.
